# Angiotensin-Converting Enzyme Inhibition as a Potential Risk Factor for Periprosthetic Joint Infection Following Total Knee Arthroplasty

**DOI:** 10.1016/j.artd.2025.101641

**Published:** 2025-02-25

**Authors:** Rishi Trikha, Nicolas Cevallos, Alan L. Zhang, Sanjiv M. Narayan, Christos Photopoulos, Alexandra Stavrakis, Nicholas M. Bernthal

**Affiliations:** a Department of Orthopaedic Surgery, University of California, Los Angeles, Los Angeles, CA, USA; b Department of Orthopaedic Surgery, University of California, San Francisco, San Francisco, CA, USA; c Department of Medicine, Stanford University Cardiovascular Institute, Stanford, CA, USA; d Department of Orthopaedic Surgery, Cedars Sinai Kerlan-Jobe Institute, Los Angeles, CA, USA

**Keywords:** Periprosthetic joint infection, Total knee arthroplasty, Angiotensin-converting enzyme inhibitors, Angiotensin receptor blockers

## Abstract

**Background:**

Periprosthetic joint infection (PJI) following total knee arthroplasty (TKA) portends significant morbidity. *In-vivo* studies demonstrating angiotensin-converting enzyme inhibitors (ACEis) may have an immunosuppressive effect. This study leveraged a large national registry to test if propensity-matched patients taking ACEis would have higher rates of PJI following TKA than patients taking angiotensin receptor blockers (ARBs).

**Methods:**

A retrospective review of the Mariner PearlDiver database was performed. Patients were divided into those taking either an ACEi or an ARB for 1 year prior to primary TKA. Irrigation and debridement and/or removal of knee prostheses procedural codes were used to identify PJI. Odds ratios (ORs) and 95% confidence intervals (CIs) were analyzed with significance defined as a *P* value < .05.

**Results:**

After propensity score matching, 39,103 patients were included in each group. The ACEi group had a higher rate of PJI compared to the ARB group at 6 months (OR: 2.69; 95% CI: 1.43-5.09; *P* < .01) and 1 year (OR: 2.94; 95% CI: 1.67-5.19; *P* < .001). The ACEi group also had higher rates of deep vein thromboses (OR: 1.33; 95% CI: 1.23-1.44), pulmonary embolisms (OR: 1.99; 95% CI: 1.73-2.30), pneumonias (OR: 1.29; 95% CI: 1.15-1.45), hematomas (OR: 1.47; 95% CI: 1.20-1.81), and transfusion (OR: 1.87; 95% CI: 1.69-2.08) within 90 days postoperatively, all *P* values < .001.

**Conclusions:**

Perioperative use of ACEi was associated with a substantially higher rate of PJI than use of ARBs. Further studies are warranted to elucidate if this represents immunosuppression or other mechanisms related to ACEi. Regardless, given the relative clinical interchangeability of ACEis and ARBs, ACEi treatment may represent an underappreciated, modifiable perioperative infectious risk factor.

## Introduction

Periprosthetic joint infection (PJI) following total joint arthroplasty is a challenging complication that portends significant morbidity. In addition to being associated with significant pain and impairment of quality of life, the 5-year survival rate of PJI for a total knee arthroplasty (TKA) is 71.7%, which is worse than breast cancer (91.2%), renal cell cancer (78.1%), or non-Hodgkin lymphoma (74.3%) [1,2]. As the number of total joint arthroplasties is expected to increase in coming years, so too are the number of PJIs [3,4]. It is estimated that by 2030, the projected national total cost for TKA PJI will be $1.1 billion annually [5]. This provides both clinical and financial motivations to develop strategies to reduce PJI.

In addition to intraoperative techniques to mitigate PJI such as systemic antibiotic administration, antibiotic-eluting implant use, and antibiotic-loaded cement use, there has been an increased emphasis on perioperative host immune optimization [[6], [7], [8], [9]]. The immune profile of a patient at the time of surgery has been shown to be associated with postoperative infection risk [9,10]. In certain instances, such as a trauma or an oncologic setting, it is not always possible to optimize immune function; however, immune function should be optimized in patients undergoing elective total joint arthroplasty.

Angiotensin-converting enzyme inhibitors (ACEis) are one of the most common antihypertensive medications among elderly patients, with 11.4% of Americans aged 40-59 years and 21.3% of Americans aged 60-79 years having taken an ACEi in the past 30 days [11]. Notably, guidelines from the American Heart Association/American College of Cardiology and the European Society of Cardiology recommend either ACEis or angiotensin receptor blockers (ARBs) as first-line antihypertensive therapy [12,13]. However, studies are beginning to indicate that treatment with ACEi diminishes immunological responses, in accordance with the increasingly recognized immunomodulatory role of ACE, which could predispose to infection [[14], [15], [16], [17]]. In contrast to ACEi, ARBs appear to favorably regulate innate and adaptive immunity [18,19].

We hypothesized that patients taking an ACEi are associated with a higher rate of PJI following TKA than patients taking an ARB. We tested our hypothesis by performing a comparison between propensity-matched patients taking either an ACEi or an ARB prior to TKA and leveraging the power of a large national database. Specifically, we sought to identify (1) the demographics of patients taking an ACEi as compared to an ARB prior to undergoing TKA, (2) whether a difference in PJI rates exists between patients taking an ACEi compared to an ARB, and (3) differences in other postoperative complications within 90 days between patients taking an ACEi compared to an ARB. There is a relative paucity of clinical evidence on this topic, which could have important clinical implications given the importance of mitigating PJI in the elderly population, who are frequently prescribed such antihypertensive medications [11,17,20,21].

## Material and methods

A retrospective analysis of the Mariner PearlDiver Database (Colorado Springs, CO; www.pearldiver.inc) was performed. The Mariner PearlDiver Database contains 122 million deidentified patient claims from self-pay, private, and government plans and is HIPAA-compliant. Given these patients were deidentified and included in a national registry, no institutional review board approval was required. The M151 Ortho subset of the Mariner PearlDiver database was used to identify patients who underwent a TKA between 2010 and 2021. Patients were separated into 2 groups: those on either an ACEi or an ARB medication as antihypertensive monotherapy for at least 1 year prior to primary TKA. Current Procedural Terminology (CPT) codes were queried to identify the patient population of interest. Irrigation and debridement CPT codes and the CPT code for the removal of knee prosthesis were used as a proxy to identify patients who developed PJI 6 months, 1 year, 2 years, and 5 years following index TKA (Table 1). Patients were not included in the appropriate data analysis if they did not have sufficient follow-up. Other postoperative complications within 90 days including deep vein thrombosis, pulmonary embolism, pneumonia, hematoma, transfusion, and wound dehiscence were also included for analysis.

**Table 1:** Current procedural terminology codes queried.

Description	CPT codes queried
Total knee arthroplasty	CPT-27447
Removal of prosthesis, including total knee prosthesis, methylmethacrylate with or without insertion of spacer, knee	CPT-27488
Incision and drainage, deep abscess, bursa, or hematoma, thigh, or knee region	CPT-27301
Incision, deep, with opening of bone cortex, femur, or knee (eg, osteomyelitis or bone abscess)	CPT-27303
Arthrotomy, knee, with exploration, drainage, or removal of foreign body (eg, infection)	CPT-27310
Arthroscopy, knee, surgical; for infection, lavage, and drainage	CPT-29871

CPT, current procedural terminology.

All statistical analyses were conducted via R statistical software (R Project for Statistical Computing, Vienna, Austria) incorporated in PearlDiver. Propensity score matching was used to minimize bias by controlling for the validated, applicable Charlson Comorbidity Index (CCI) [22,23]. The nearest-neighbor method was incorporated to match patients from the ACEi group in a 1:1 ratio with patients in the ARB group in the closest propensity score in descending order. The quality of matching was quantified using the standardized mean difference with groups considered more balanced across a given covariate if the standardized mean difference was less than 0.1, which is common standard in propensity score matching studies [24]. Unmatched patients from the control group were not included in further statistical analysis of outcome measures. Kaplan-Meier survival curves and log-rank test were used to calculate survival differences of irrigation and debridement procedures up to 10 years from index surgery. Survival probability was defined as the probability of not developing PJI. Odds ratios (ORs) and 95% confidence intervals (CIs) were analyzed with significance defined as a *P* value of < .05.

## Results

### Demographics

A total of 116,936 patients who underwent TKA and were taking an ACEi or ARB medication between 2010 and 2021 were included for analysis. There were 77,831 patients in the ACEi group, and 39,105 in the ARB group prior to propensity score matching. The median age in the ACEi group was 67 years (interquartile range: 61-73), while the median age in the ARB group was 69 years (interquartile range: 63-74). Women comprised 43.6% and 34.6% of patients in the ACEi and ARB groups, respectively. The mean CCI score in the ACEi and ARB groups was 1.95 ± 2.18 and 2.06 ± 2.18 (*P* < .01), respectively. There were more patients with diabetes in the ACEi group (59.6%) when compared to the ARB group (57.2%, *P* < .0001). There were also more patients with coronary artery disease (44.6% vs 42.3%) and chronic kidney disease (30.7% vs 29.6%) in the ACEi compared to the ARB groups, with all *P* values < .0001 (Table 2).

**Table 2:** Baseline patient comorbidities prepropensity score matching of patients taking an ACEi vs patients taking an ARB.

Patient comorbidities	ACEi (n = 77,831)		ARB (n = 39,105)		*P* value
	N	%	N	%	
Diabetes	46,420	59.6	22,355	57.2	**<.0001**
Obesity	46,716	60.0	24,510	62.7	**<.0001**
Coronary artery disease	34,702	44.6	16,554	42.3	**<.0001**
Chronic kidney disease	23,932	30.7	11,582	29.6	**<.0001**
Rheumatoid arthritis	5482	7.0	2785	7.1	.6303

ACEi, angiotensin-converting enzyme inhibitor; ARB, angiotensin receptor blocker.

Bold values indicate statistical significance (*P* value < .05).

### Periprosthetic joint infection rates

**Figure 1:**
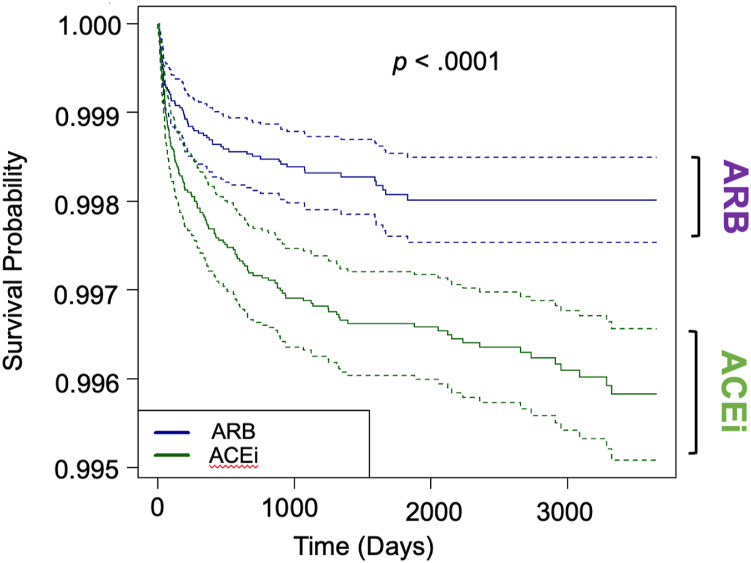
Kaplan-Meier survival curve comparing patients taking an ACEi compared to patients taking an ARB. Survival probability defined as probability of no infection. 95% confidence interval depicted. ACEi, angiotensin-converting enzyme inhibitor; ARB, angiotensin receptor blocker.

After propensity score matching, 39,103 patients were assigned to each antihypertensive group. The CCI in the ACEi group was 2.06 ± 2.17 and in the ARB group was also 2.06 ± 2.17 (*P* = 1). The median was 1 and the interquartile range was 3 for both groups. The ACEi group had a higher rate of PJI compared to the ARB group at 6 months following index surgery with an OR of 2.69, 95% CI: 1.43-5.09 (*P* < .01). At 1 year after index surgery, the ACEi group also had a higher rate of PJI compared to the ARB group with an OR of 2.94, 95% CI: 1.67-5.19 (*P* < .001). At 2 years after index surgery, the OR was 2.95, 95% CI: 1.78-4.90 (*P* < .0001), and at 5 years after index surgery, the OR was 2.64, 95% CI: 1.71-4.09 (Table 3, *P* < .0001). When defining survival probability as not undergoing a subsequent irrigation and debridement procedure, the Kaplan-Meier survival curve demonstrated that patients taking an ACEi had a significantly lower survival rate compared to patients taking ARB (Fig. 1, *P* < .0001).

**Table 3:** Periprosthetic joint infection risk of patients taking an ACEi vs patients taking an ARB after propensity score matching.

Time from index surgery	ACEi (n = 39,103)		ARB (n = 39,103)		ACEi odds ratio (95% confidence interval)	*P* value
	N	%	N	%		
6 months	35	0.09	13	0.03	2.69 [1.43, 5.09]	<.01
1 year	47	0.12	16	0.04	2.94 [1.67, 5.19]	<.001
2 years	59	0.15	20	0.05	2.95 [1.78, 4.90]	<.0001
5 years	74	0.19	28	0.07	2.64 [1.71, 4.09]	<.0001

ACEi, angiotensin-converting enzyme inhibitor; ARB, angiotensin receptor blocker.

### Postoperative complications within 90 days

After propensity score matching, there were higher rates of patients suffering a variety of complications within 90 days postoperatively in the ACEi group compared to the ARB group. These included deep vein thrombosis (OR: 1.33; 95% CI: 1.23-1.44), pulmonary embolism (OR: 1.99; 95% CI: 1.73-2.30), pneumonia (OR: 1.29; 95% CI: 1.15-1.45), hematoma (OR: 1.47; 95% CI: 1.20-1.81), urinary tract infections (OR: 1.16; 95% CI: 1.08-1.24), or transfusion (OR: 1.87; 95% CI: 1.69-2.08) with all *P* values < .001 (Table 4).

**Table 4:** Postoperative complications within 90 days of propensity score-matched cohorts of patients taking an ACEi vs patients taking an ARB.

Patient comorbidities	ACEi (n = 39,103)		ARB (n = 39,103)		ACEi odds ratio (95% confidence interval)	*P* value
	N	%	N	%		
Deep vein thrombosis	1493	3.82	1135	2.90	1.33 [1.23, 1.44]	**<.0001**
Pulmonary embolism	588	1.50	297	0.76	1.99 [1.73, 2.30]	**<.0001**
Pneumonia	629	1.61	489	1.25	1.29 [1.15, 1.45]	**<.0001**
Hematoma	226	0.58	154	0.39	1.47 [1.20, 1.81]	**<.001**
Transfusion	1079	2.76	583	1.49	1.87 [1.69, 2.08]	**<.0001**
Wound dehiscence	447	1.14	422	1.08	1.06 [0.93, 1.20]	0.413

ACEi, angiotensin-converting enzyme inhibitor; ARB, angiotensin receptor blocker.

Bold values indicate statistical significance (*P* value < .05).

## Discussion

PJI is a devastating complication of TKA that imparts significant morbidity and is associated with a large cost to the healthcare system [25,26]. Thus, techniques to optimize the host immune response to prevent PJI are of great importance. Given the relatively high number of elderly patients taking an ACEi or an ARB in conjunction with the projected increase in the annual number of total joint arthroplasties, the risk profiles of these 2 agents were studied [11,27,28]. The present study found that patients undergoing a TKA who were taking an ACEi perioperatively were at an increased risk of developing PJI as well as other complications when compared to patients who were taking an ARB.

### PJI risk when comparing ACEis and ARBs

The present study demonstrated that propensity-matched patients treated with an ACEi had a higher risk of developing PJI than patients treated with an ARB. This risk was statistically significant at 6 months, 1 year, 2 years, and 5 years from index surgery. Prior in-vivo studies suggest that the purported mechanisms behind angiotensin-converting enzyme (ACE) inhibition exerting a possible immunosuppressive effect include a decrease in proinflammatory cytokines, dysfunctional macrophage activity, and/or impaired chemotaxis [16,17,29,30]. Other in-vivo studies showed that neutrophils expressing increased ACE levels in transgenic mice had enhanced antibacterial function [31]. In a mouse model of PJI from the authors’ study group, mice treated with an ACEi as compared to an ARB demonstrated higher rates and severity of PJI. The mechanism behind this was decreased function of monocytes and neutrophils as well as decreased recruitment of monocytes and neutrophils [20]. The present study supports the notion that treatment with an ACEi as compared to an ARB could potentially have an immunosuppressive effect.

The present study was not equipped to comment on the PJI risk after switching from an ACEi to an ARB. However, when considering switching from an ACEi to an ARB, it should be noted that the European Society of Cardiology suggests that it is feasible to stop an ACEi and start an ARB the following day [32]. Another study analyzing a new class of drugs called angiotensin receptor neprilysin inhibitors indicated that, to reduce the risk of angioedema, a wash-out period of 36 hours was applied between the last dose of an ACEi and the initiation of another antihypertensive [33].

### Other surgical complications when comparing ACEis and ARBs

This study found a higher rate of pneumonia at 90 days postoperatively in the ACEi group when compared to the ARB group. A study conducted by Silva-Santos et al. demonstrated that ACEi treatment increased lung ACE 2 levels which subsequently increased viral loads in patients with COVID-19 [34]. While this did not necessarily translate to higher disease severity, it is possible that the same mechanism is responsible for the increased rates of pneumonia in the ACEi group seen in the present study. Moreover, through a similar mechanism, men hospitalized with acute COVID-19 who were on ARB treatment had better outcomes than men not on ARBs [35].

This study also found higher rates of hematomas at 90 days postoperatively in the ACEi group when compared to the ARB group. One possible rationale is the induced bradykinin elevation causing increased vascular permeability. This was the rationale proposed by Neidert et al. where ACEi treatment was associated with higher hematoma volume in patients with chronic subdural hematomas [36]. The present study also found higher rates of transfusions in the ACEi group. One possible rationale would be that the higher rate of hematomas could potentially lead to higher rates of transfusions.

It is unclear as to why the ACEi group in this study demonstrated higher rates of deep vein thromboses and pulmonary embolisms when compared to the ARB group. The use of both ACEi and ARBs has been shown to have possible antithrombotic effects via improvement of blood rheology, thus obviating venous stasis [37]. However, a multinational cohort study by Chen et al. demonstrated no significant differences between ACEi and ARB treatment in the development of deep vein thromboses or pulmonary embolisms [38].

### Choosing an ARB over an ACEi

The first joint recommendation on the pharmacologic treatment of hypertension from the American Heart Association and the American College of Cardiology in 2013 indicated that either ACEis or ARBs were appropriate first-line agents. Each subsequent recommendation has also stated that ACEis or ARBs are appropriate first-line agents, with no preference given to either medication [39]. Despite this, the present study also shows that the number of patients taking an ACEi prior to a TKA was almost double the number of patients taking an ARB. It is possible that the rationale for this is that patients have historically been placed on an ACEi. Prior to 1995, advantages of ACEis over ARBs were availability and cost. However, with the introduction of generic ACEis and ARBs in 2010, the utilization and cost of ACEis and ARBs have become similar, with some generic ARBs now costing less than ACEis [40].

Another argument to use ARBs rather than ACEis stems from the safety profile of both drugs. While ACEis were historically preferentially used in patients with diabetes and kidney disease, Chen et al. demonstrated that ARBs do not differ statistically significantly in effectiveness compared to ACEis. ARBs did, however, present a better safety profile with significantly lower risks of angioedema, cough, pancreatitis, and gastrointestinal bleeding [38]. The baseline patient comorbidities prepropensity score matching in the present study demonstrating more patients with diabetes and kidney disease in the ACEi group may be due to historical preferential use in these patient populations. More recently, Li et al. demonstrated that patients taking an ACEi also had a higher incidence of patients stopping their medication due to adverse events when compared to ARBs [41]. When looking at the market share of the total prescriptions of ACEis vs ARBs over the past 20 years, the trend has shifted from prescribers favoring ACEis to not favoring either medication to now slightly favoring ARBs [40]. This may be due to prescribers noting the increased adverse effects of ACEis while acknowledging recent hypertension guidelines that the efficacy of ACEis and ARBs are comparable [42]. The present study found that the CCI was actually higher in the ARB group in our prematched cohort. One possible rationale for this is prescribers understanding the importance of the better safety profile of ARBs in patients with more comorbidities.

There is a surge of cardiologic literature suggesting the preferred use of ARBs over ACEis. In addition to the difference in safety profile, this surge is predicated on the difference in efficacy between the 2 drugs in terms of a surrogate endpoint of blood pressure, but also for outcomes such as coronary artery disease, stroke, renoprotection, and all-cause mortality [[42], [43], [44]]. Furthermore, there may be a generational gap between trials evaluating the efficacy of ARBs and ACEis [42,43]. The present study adds to the growing body of literature favoring the use of ARBs over ACEis.

### Limitations

There are several limitations to the present study in addition to the expected shortcomings of using a national database. Given that this study uses CPT codes, there is an inherent possibility of inaccurate coding and/or data entry. The use of a CPT code also limits diagnostic detail and mechanistic analysis. This does not allow for analysis of certain variables such as the microorganism causing PJI, if there was any potential delay in surgical treatment, type of implant/spacer used, or PJIs that were treated nonoperatively. Furthermore, given CPT codes are procedural in nature, patients who developed an infection that were treated nonoperatively with antibiotics were not able to be analyzed. The lack of diagnostic detail also pertains to the type of medications that were used. In this sense, there is no way to determine if any differences exist among different ACEis or ARBs nor any way to stratify based on severity of specific comorbidities. The present study is thus not equipped to comment on the optimal timing of changing a patient from an ARB to an ACEi or whether there is any sort of washout period. Additionally, there is lack of follow-up data to determine if any differences existed in resolution of infection, patient satisfaction, or postoperative function. Despite these limitations, this is the first clinical study of its kind to analyze the infectious ramifications of ACEi use as compared to ARB use. Prospective studies are certainly warranted to further tease out the perioperative infectious risk of these antihypertensives.

## Conclusions

The present study, in addition to the authors’ prior mouse model of PJI, suggests that the use of ACEis may potentially portend an increased perioperative infectious risk when compared to the use of ARBs [20]. This potential perioperative risk adds to the recent body of clinical evidence supporting the use of ARBs over ACEis [38,41,42,[45], [46], [47]]. Given the relative interchangeability of ACEi and ARBs, ACEi treatment may represent an underappreciated, modifiable perioperative infectious risk factor.

## Conflicts of interest

All authors have no financial or other conflicts of interest pertinent to this work.

For full disclosure statements refer to https://doi.org/10.1016/j.artd.2025.101641.

## CRediT authorship contribution statement

**Rishi Trikha:** Writing – review & editing, Writing – original draft, Visualization, Validation, Supervision, Software, Resources, Project administration, Methodology, Investigation, Funding acquisition, Formal analysis, Data curation, Conceptualization. **Nicolas Cevallos:** Writing – original draft, Validation, Methodology, Investigation, Formal analysis, Data curation, Conceptualization. **Alan L. Zhang:** Writing – review & editing, Methodology, Investigation, Formal analysis, Data curation, Conceptualization. **Sanjiv M. Narayan:** Writing – review & editing, Writing – original draft, Visualization, Supervision, Project administration, Methodology, Investigation, Conceptualization. **Christos Photopoulos:** Writing – review & editing, Writing – original draft, Supervision, Software, Methodology, Investigation, Formal analysis, Data curation, Conceptualization. **Alexandra Stavrakis:** Writing – review & editing, Writing – original draft, Validation, Supervision, Project administration, Methodology, Investigation, Formal analysis, Data curation, Conceptualization. **Nicholas M. Bernthal:** Writing – review & editing, Writing – original draft, Supervision, Project administration, Methodology, Investigation, Formal analysis, Data curation, Conceptualization.

## Appendix A Supplementary data

Conflict of Interest Statement for Bernthal

Conflict of Interest Statement for Cevallos

Conflict of Interest Statement for Narayan

Conflict of Interest Statement for Photopoulos

Conflict of Interest Statement for Stavrakis

Conflict of Interest Statement for Trikha

Conflict of Interest Statement for Zhang
